# *Sarcandra glabra* (Caoshanhu) protects mesenchymal stem cells from oxidative stress: a bioevaluation and mechanistic chemistry

**DOI:** 10.1186/s12906-016-1383-7

**Published:** 2016-10-28

**Authors:** Jingjing Liu, Xican Li, Jian Lin, Yunrong Li, Tingting Wang, Qian Jiang, Dongfeng Chen

**Affiliations:** 1 School of Chinese Herbal Medicine, Guangzhou University of Chinese Medicine, Waihuan East Road No.232, Guangzhou Higher Education Mega Center, 510006 Guangzhou, China; 2 School of Basic Medical Science, Guangzhou University of Chinese Medicine, Guangzhou, China 510006

**Keywords:** Antioxidant mechanism, Astilbin, Caoshanhu, Electron transfer, Fe-chelating, Hydrogen atom transfer, Mesenchymal stem cells, Rosmarinic acid, *Sarcandra glabra*

## Abstract

**Background:**

*Sarcandra glabra* (Caoshanhu) is a traditional Chinese herbal medicine used for treating various oxidative-stressed diseases. The present work evaluated its protective effect on mesenchymal stem cells (MSCs) from oxidative stress and then discussed possible mechanisms underlying this observation.

**Methods:**

Ethanolic extract of *S. glabra* (ESG) was investigated by chemical methods for its content of total phenolics, rosmarinic acid, and astilbin. ESG, along with rosmarinic acid and astilbin, was investigated for the effect on the viability of Fenton-treated MSCs using 3-(4,5-Dimethylthiazol-2-yl)-2,5-diphenyl (MTT) assay. The observed cell protective effect was further explored by mechanistic chemistry using various antioxidant assays, including DNA protection, •OH-scavenging, •O_2_^−^-scavenging, FRAP (ferric ion reducing antioxidant power), ABTS^+^•-scavenging, DPPH•-scavenging, and Fe^2+^-chelating assays.

**Results:**

Analysis of ESG revealed a content of 46.31 ± 0.56 mg quercetin/g total phenolics, 0.78 ± 0.01 % rosmarinic acid, and 3.37 ± 0.01 % astilbin. Results from the MTT assay revealed that three compounds (rosmarinic acid>astilbin>ESG) could effectively increase the survival of Fenton-treated MSCs. Similarly, in •O_2_^−^-scavenging, DPPH•-scavenging, and Fe^2+^-chelating assays, rosmarinic acid exhibited more activity than astilbin; while in FRAP, ABTS^+^•-scavenging assays, astilbin was stronger than rosmarinic acid.

**Conclusion:**

*S. glabra* can prevent MSCs from •OH-induced oxidative stress. Such protective effect can be attributed to its antioxidant ability and the presence of two kinds of phytophenols, i.e. caffeoyl derivatives and flavonoids. As the respective representatives of caffeoyl derivatives and flavonoids, rosmarinic acid and astilbin may exert the antioxidant action via direct ROS-scavenging and indirect ROS-scavenging (i.e. Fe^2+^-chelating). The direct ROS-scavenging ability involves hydrogen atom transfer (HAT) and/or electron transfer (ET) pathway. Astilbin engages the latter pathway more, which can be attributed to the larger planar conjugation in A/C fused rings. Rosmarinic acid, on the other hand, shows more HAT and Fe^2+^-chelating potential, which may be due to rosmarinic acid bearing one more catechol moiety whereas astilbin has steric-hindrance from 3-α-L-rhamnose and an H-bonding between 4,5 sites. The antioxidant features of rosmarinic acid can be generalized to other caffeoyl derivatives, while that of astilbin cannot be generalized to other flavonoids because of the difference in chemical structures.

**Electronic supplementary material:**

The online version of this article (doi:10.1186/s12906-016-1383-7) contains supplementary material, which is available to authorized users.

## Background

Owing to the ease of isolation, manipulability, and potential for differentiation, mesenchymal stem cells (MSCs) are of great interest to clinicians for their great potential to enhance tissue engineering for the treatment of various diseases [1], especially neurodegenerative diseases [2], osteoarthritis [3], and cancers [4]. However, during the process of proliferation and differentiation, chemical or physical stimuli, such as radiation [5] and iron overload [6], can generate the •OH radical to cause oxidative stress-induced apoptosis of these cells. This poor viability has prevented the clinical application of the transplantation of MSCs.

In fact, in autologous stem cell transplantation for cancer patients, radiotherapy has been recently indicated to decrease cell survival [7]. The most recent study pointed out that MSCs can even promote tumor recurrence after stereotactic body radiation therapy [8]. These are believed to be related to the oxidative stress induced by reactive oxygen species ROS, especially •OH (the most toxic form of ROS) [9].

**Fig. 1:**
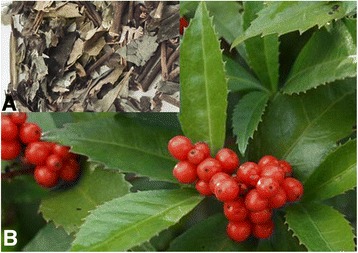
The photos of Caoshanhu (**a**) and the plant of *Sarcandra glabra* (Thunb.) Nakai (**b**)

Interestingly, traditional Chinese Medicine (TCM) views this oxidative-stressed apoptosis, as well as the dysfunction of viability, proliferation, and differentiation, as a syndrome arising from so-called *heat-toxic*. The *heat-toxic* can be countered by a great deal of Chinese herbal medicines, such as Caoshanhu (or Zhongjiefeng, Fig. 1a).

Caoshanhu is the dried whole plant of *Sarcandra glabra* (Thunb.) Nakai (*S. glabra*, Fig. 1b), which is a shrub widely distributed in China and other Asian countries. In TCM, *S. glabra* is frequently used to treat various diseases relevant to heat-toxic, especially pneumonia, epidemic encephalitis B, appendicitis, shigellosis, and cancers [10]. All of these diseases however have been suggested to be linked to oxidative stress, in free radical biology and medicine [11]. This implies that *S. glabra* will be able to play a role in repairing apoptosis of MSCs in the transplantation process.

Phytochemical study has shown that, *S. glabra* contains at least 50 components that can be classified into five types: organic acids, caffeoyl derivatives, flavonoids, coumarins, and terpenoids [12, 13]. Caffeoyl derivatives and flavonoids were newly reported to be the first main bioactive compounds; while isofraxidin (a coumarin) was considered as the second main bioactive compound [14]. As such, rosmarinic acid (RA) and astilbin (AS) were selected as the two typical bioactive compounds in the present study. It is worth noting that isofraxidin is utilized as the “marker compound” in Zhongjiefeng Tablet in *Chinese Pharmacopoeia* [10]. However, isofraxidin is actually less relevant to the present study.

Consequently, in present study we comparatively investigated the effects of *S. glabra*, RA, and AS toward the viability of oxidative-stressed MSCs, then further discussed the possible mechanistic chemistry based on the structure-activity relationship of RA and AS. This approach will help understand the beneficial effects of *S. glabra* as a Chinese Herbal medicine, as well as support the screening of natural phytophenols and their synthetic derivatives as effective antioxidants for cell transplantation purposes.

## Methods

### Plant and animals

Caoshanhu (LOT. 150210311) was purchased from Kangmei Pharmaceutical Co. Ltd (Shantou, China) and authenticated by Professor Shuhui Tan. Sprague–Dawley (SD) rats of 4 weeks of age were obtained from the Animal Center of Guangzhou University of Chinese Medicine.

### Chemicals

Rosmarinic acid (CAS 20283-92-5, 98 %) was purchased from Shanghai Aladdin Chemistry Co., Ltd. (Shanghai, China); Astilbin (CAS 29838-67-3, 98 %) was from Chengdu Biopurify Phytochemicals Ltd. (Chengdu, China). Dulbecco’s modified Eagle’s medium (DMEM) and fetal bovine serum (FBS) were purchased from Gibco, Inc. (Grand Island, NY, USA). CD44 was from Wuhan Boster Co., Ltd. (Wuhan, China). DPPH• (1,1-diphenyl-2-picryl-hydrazl), neocuproine (2,9-dimethyl-1,10-phenanthroline), BHA (butylated hydroxyanisole), Trolox [(±)-6-hydroxyl-2,5,7,8-tetramethlychromane-2-carboxylic acid], Ferrozin [3-(2-pyridyl)-5,6-bis(4-phenylsulfonicacid)-1,2,4-triazine], Percoll system, and pyrogallol were obtained from Sigma-Aldrich Trading Co. (Shanghai, China); ABTS [2,2′-azino-bis(3-ethylbenzo-thiazoline-6-sulfonic acid diammonium salt)] and D-2-deoxyribose were from Amresco Chemical Co. (Solon, OH, USA); DNA sodium salt (fish sperm) was purchased from Aladdin Chemistry Co. (Shanghai, China); Acetonitrile was purchased from Merck Serono Co., Ltd. (Shanghai, China); Acetonitrile and water were of HPLC grade. All other reagents were of analytical grade.

### Preparation of ethanol extract of *S. glabra* (ESG)

The preparation of ethanol extract of *S. glabra* was based on the method [15]. In brief, the dried *S. glabra* (Caoshanhu) was ground into coarse powder then extracted with refluxing method using 18-fold ethanol for 6 h. The extract was filtered using Büchner funnel and filter paper. The ethanol extract was concentrated to dryness under reduced pressure at 60 °C using a rotary evaporator. The dried extract was named ethanol extract of *S. glabra* (ESG) and stored at 4 °C for further analysis.

### Determination of total phenolics

The total phenolics of the ESG were determined using a modified Folin-Ciocalteu colorimetric method [16, 17]. In brief, 0.1 mL ESG methanolic solution (1 mg/mL) was mixed with 0.5 mL Folin-Ciocalteu reagent (0.25 mol/L). The mixture was left standing for 3 min, followed by the addition of Na_2_CO_3_ aqueous solution (1.0 mL, 15 %, *w/v*). After standing at room temperature for 30 min, the mixture was centrifuged at 3500 *g*/min for 3 min. The absorbance of the supernatant was measured at 760 nm (Unico 2100, Shanghai, China). The determinations were performed in triplicate, and the calculations were based on a calibration curve obtained with quercetin, the linear regression equation was *y* = 0.1296*x* + 0.0848 (*x* for quercetin content, *y* for absorbance at 760 nm, *R* = 0.998). The result was expressed as quercetin equivalents in milligrams per gram of extract.

### HPLC analysis for RA and AS in ESG

HPLC analysis was performed on Waters e2695 (Los Angeles, California, USA) equipped with Agilent 5 TC-C_18_ (250 mm × 4.6 mm, 5 μm) (Beijing, China). The mobile phase consisted of acetonitrile (A)-0.5 % trifluoroacetic acid in water (B) (0~10 min, remain 15 % A; 10~50 min, 15 % A~25 % A; 50~80 min, 25 % A~80 % A; 80~85 min, 80 % A~15 % A), the flow rate was 1.0 mL/min, injection volume was 20 μL and absorption was measured at 254 nm [18]. In the study, RA and AS in ESG were identified by comparing their retention times and the peak areas were employed to characterize the relative contents of RA and AS using the linear regression equations *y* = 1480.4*x* + 406,988 (*R* = 0.988) and *y* = 22753*x* + 462,876 (*R* = 0.962), respectively.

### Protecting MSCs against oxidative stressed apoptosis (MTT assay)

The MSCs were cultured according to a slightly modified version of the methods described in our previous report [19]. Briefly, bone marrow samples were obtained from the femurs and tibias of rats, and the resulting samples were diluted with DMEM (LG: low glucose) containing 10 % FBS. The MSCs were obtained by gradient centrifugation at 900 *g*/min for 30 min on a 1.073 g/mL Percoll system. The cells were then detached by treatment with 0.25 % trypsin and passaged into culture flasks at a density of 1 × 10^4^ cells/cm^2^. The homogeneity of the MSCs was evaluated at passage 3 based on their CD44 expression by flow cytometry. These cells were then used for the following experiments.

These MSCs were seeded into 96-well plates (4 × 10^3^ cells/well). After adherence for 24 h, the cells were divided into three groups, including control, model and samples groups. The MSCs in the control group were incubated for 24 h in DMEM. The MSCs in the model group were injured for 1 h using FeCl_2_ (100 μM) followed by H_2_O_2_ (50 μM). The resulting mixture of FeCl_2_ and H_2_O_2_ was removed and the MSCs were incubated for 24 h in DMEM. The MSCs in the samples groups were injured and incubated for 24 h in DMEM in the presence of various concentrations of samples. After being incubated, the cells were treated with 20 μL of MTT (5 mg/mL in PBS), and the resulting mixtures were incubated for 4 h. The culture medium was subsequently discarded and replaced with 150 μL of DMSO. The absorbance of each well was then measured at 490 nm using a Bio-Kinetics plate reader (PE-1420; Bio-Kinetics Corporation, Sioux Center, IA, USA). The serum medium was used for the control group and each sample test was repeated in five independent wells.

### Mechanistic chemistry experiments

Mechanistic chemistry experiments mainly included various antioxidant assays, e.g. DNA protection assay, •OH scavenging (deoxyribose degradation) assay, •O_2_^−^ scavenging (pyrogallol autoxidation) assay, ABTS^+^• scavenging assay, DPPH• scavenging assay, and Fe^2+^-chelating assay. Among them, the former three methods have been established by our team [20–22]. The latter three methods were described in our previous paper [23]. In addition, FRAP (ferric ion reducing antioxidant power) assay was also performed in pH 3.6 buffer [24]. On the basis of the relevant formulas, the dose response curves were plotted to calculate IC_50_ values (in μg/mL). The IC_50_ values were then transferred into ones in molar unit (μM, Table 1). The detailed experimental protocols are shown in the additional file (Additional file 1).

**Table 1:** The IC_50_ values of ESG, rosmarinic acid (RA), astilbin (AS), and the positive controls

Assays	ESG μg/mL	RA μg/mL (μM)	AS μg/mL (μM)	Positive controls	
				Trolox μg/mL (μM)	BHA μg/mL (μM)
DNA protective effect	79.9 ± 10.4	44.9 ± 8.9 (126.7 ± 23.1^b^)	70.9 ± 1.6 (193.3 ± 5.8^c^)	85.0 ± 21.3 (85.0 ± 21.3^a^)	67.8 ± 12.4 (376.3 ± 69.2^d^)
•OH scavenging	109.5 ± 4.5	100.7 ± 0.9 (280.0 ± 1.0^b^)	97.9 ± 5.5 (220.0 ± 10.0^a^)	110.0 ± 4.6 (441.0 ± 20.1^d^)	63.3 ± 7.8 (353.3 ± 41.6^c^)
•O_2_^−^ scavenging	140.8 ± 3.1	18.4 ± 0.4 (50 ± 0.4^a^)	132.8 ± 3.4^b^ (295.0 ± 7.5^b^)	167.3 ± 11.1 (668.3 ± 40.2^c^)	146.6 ± 4.4 (813.0 ± 24.0^d^)
FRAP	24.9 ± 1.0	7.1 ± 1.2 (19.6 ± 3.2^b^)	5.4 ± 0.2 (12.0 ± 0.4^a^)	6.9 ± 0.3 (27.6 ± 1.2^c^)	4.4 ± 0.2 (24.0 ± 1.1^c^)
ABTS^+^• scavenging	12.1 ± 1.8	6.8 ± 0.4 (18.7 ± 2.1^b^)	5.0 ± 0.7 (11.1 ± 1.7^a^)	7.2 ± 0.1 (29.0 ± 0.1^c^)	5.9 ± 0.1 (33.3 ± 0.1^c^)
DPPH• scavenging	48.6 ± 0.3	1.3 ± 0.1 (4.0 ± 0.1^a^)	8.7 ± 0.2 (19.7 ± 5.8^d^)	1.5 ± 0.1 (6.2 ± 0.1^b^)	1.9 ± 0.1 (10.8 ± 0.3^c^)
Fe^2+^ chelating	134.2 ± 15.6	128.1 ± 10.4 (355.7 ± 29.1^b^)	218.8 ± 11.0 (485.0 ± 21.2^c^)	63.9 ± 4.4 (217.3 ± 15.3^a^)^d^	ND

IC_50_ value is defined as the concentration of 50 % effect percentage and expressed as mean ± SD (*n* = 3). Means values with different superscripts (a, b) in the same row are significantly different (*p* < 0.05), while with same superscripts are not significantly different (*p* < 0.05). ^d^The positive control is sodium citrate. *BHA* butylated hydroxyanisole, *ESG* Ethanolic extract of *Sarcandra glabra (Thunb.) Nakai*, *ND* Cannot be detected, *FRAP* ferric ion reducing antioxidant power

### Statistical analysis

The IC_50_ values were calculated by linear regression analysis. All linear regression in this paper was analyzed by Origin 6.0 professional software. Determination of significant differences between the mean IC_50_ values of the sample and positive controls was performed using one-way ANOVA the *T*-test. The analysis was performed using SPSS software 13.0 (SPSS Inc., Chicago, IL) for windows. *P* < 0.05 was considered to be statistically significant.

## Results and discussion

**Fig. 2:**
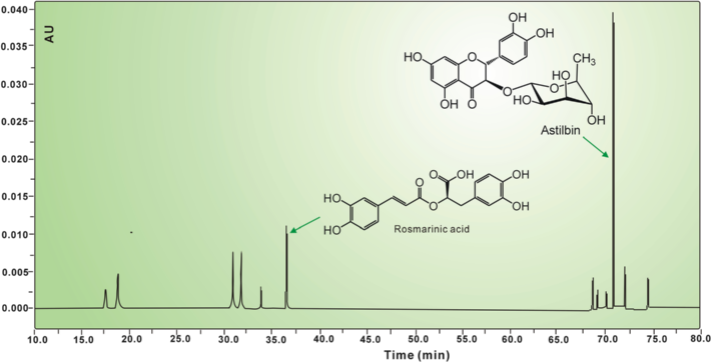
A typical HPLC profile of ESG (ethanol extract of *Sarcandra glabra*)

In the study, we first determined the total phenolics content of ESG using Folin-Ciocalteu reagent. The data suggested a high level of total phenolics (46.31 ± 0.56 mg quercetin/g) in ESG. The total phenolics mainly include caffeoyl derivatives and flavonoids, as mentioned above. In our study, a typical caffeoyl derivative RA and a flavonoid AS were successfully detected in ESG using HPLC (Fig. 2). The contents of RA and AS were 0.78 ± 0.01 % and 3.37 ± 0.01 %, respectively. In subsequent experiments, ESG along with RA and AS were investigated for their beneficial effect on oxidative-stressed MSCs.

**Fig. 3:**
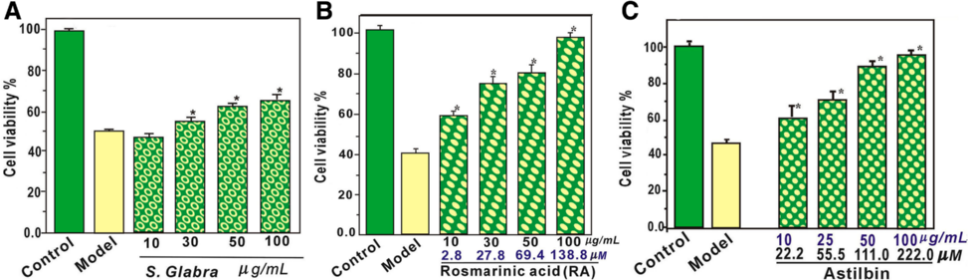
Ethanolic extract of *Sarcandra glabra* (ESG, **a**), rosmarinic acid (RA, **b**), and astilbin (AS, **c**) prevent MSCs against Fenton-induced apoptosis. Cell viability was assessed using the MTT method. Experiments were performed with 3 different batches of cells and each batch was tested in triplicate. Data are the mean ± SD values. (*) *p* < 0. 05, compared with MSCs damage following FeCl_2_ *plus* H_2_O_2_

As mentioned above, iron overload can cause oxidative stress-induced apoptosis [5], because it can yield •OH radicals through Fenton reaction (Eq. 1). Therefore, we used Fenton reagent (i.e. FeCl_2_ *plus* H_2_O_2_) as the •OH radical generator for the study. As illustrated in Fig. 3a, ESG at 10–100 μg/mL could efficiently increase the viability of MSCs treated by Fenton reagent. This implies that ESG could protect MSCs from oxidative stress-induced apoptosis. Under the same concentrations, interestingly, RA and AS exhibited a better protective effect than ESG (Fig. 3). The findings might partially explain the beneficial effects of Caoshanhu on various diseases related to *heat-toxic* in TCM and support the rationality of RA and AS as two typical bioactive compounds. More importantly, these observations also suggest RA and AS as two good candidates for transplantation therapy based on MSCs.

1 $$ {\mathrm{Fe}}^{2+}+{\mathrm{H}}_2{\mathrm{O}}_2\to {\mathrm{Fe}}^{3+}+\bullet \mathrm{O}\mathrm{H}+{\mathrm{O}\mathrm{H}}^{-} $$

**Fig. 4:**
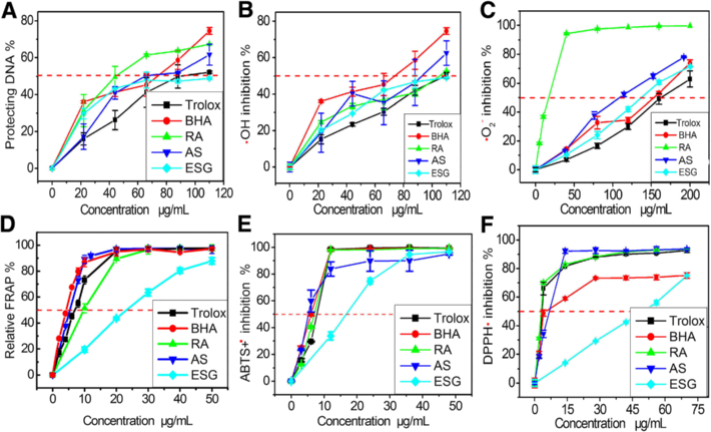
The dose response curves of ESG, rosmarinic acid (RA), and astilbin (AS) in a series of antioxidant assays: (**a**) protective effect against DNA damage; (**b**) hydroxyl (•OH) radical-scavenging; (**c**) superoxide anion (•O_2_^−^) radical-scavenging assay; (**d**) FRAP (ferric ion reducing antioxidant power); (**e**) ABTS•^+^ radical-scavenging assay; and (**f**) DPPH radical-scavenging assay. Each value is expressed as mean ± SD (*n* = 3); *ESG* ethanol extract of *Sarcandra glabra*, Trolox and BHA (butylated hydroxyanisole) were used as the positive controls

Oxidative-stressed apoptosis is suggested to closely associate with •OH-induced DNA oxidative lesions (e.g. 8-hydroxy-2′-deoxyguanosine and 8-oxo-7, 8-dihydroguanine) [25, 26]. Accordingly, we assayed its protection on DNA using a previously described approach [20]. As seen in Fig. 4a, ESG, RA, and AS (at 20–110 μg/mL) effectively prevented •OH-mediated DNA damage. This is consistent with the previous report that ESG, RA and AS played crucial roles in anti-cancer [27, 28], because carcinogenesis has been demonstrated to arise from oxidative stress. Similar results can also be observed in the •OH radical-scavenging assay based on deoxyribose degradation (Fig. 4b), where ESG, RA, and AS increased their •OH radical-scavenging activities in a concentration-dependent fashion. The similarity of the dose response curves between Fig. 4a and b indicated that their protection on MSCs and DNA were mainly based on ROS scavenging (especially •OH radical-scavenging). ESG, RA, and AS exhibited slight differences (Fig. 4a and b) because the •OH radical with its extreme reactivity can damage all types of chemical structures.

However, in the •O_2_^−^ radical scavenging assay, we observed a significant (*p* < 0.05) difference among ESG, RA, and AS (Fig. 4c). As shown in Table 1, their relative •O_2_^−^-scavenging levels decreased in the order of RA>AS>ESG. This order can better reflect the relative ROS-scavenging levels among the three samples, because •O_2_^−^ radical is a milder and typical form of ROS. However, it is worth mentioning that, the previous data about the •O_2_^−^-scavenging of AS were incorrect, because the researchers used alkaline buffer (e.g. pH 10 [29], pH 8.0 [30]) for the investigation. Under such alkaline condition, ionization of phenolic –OH as acid would predominate the chemical action to generate H^+^ and PhO^−^. PhO^−^, however, underwent electron-donating inductive effect (+I) to enhance the next O-H bond in phenolic –OH to lessen the possibility of its homolysis, then to reduce the radical-scavenging level [21, 31]. Thus, abnormal dose response curves were observed in the previous study, in which the •O_2_^−^-scavenging level of AS was even lower than that of a plant extract containing AS [30].

The difference of the three samples in •O_2_^−^ scavenging level is assumed to be linked to the antioxidant mechanisms and their chemical structures. In the aspect of mechanistic chemistry, both •O_2_^−^ scavenging and •OH scavenging are considered to be mediated through electron transfer (ET) and hydrogen atom transfer (HAT, or hydrogen-donating) [31–34].

To examine the possibility of ET, we analyzed ESG, RA, and AS by FRAP. The data suggested that they can reduce Fe^3+^ to Fe^2+^ with high efficiency (Fig. 4d). This assay was conducted under acidic condition wherein the ionization of H^+^ was thus suppressed by environment and only ET can take place [35]. Our data in Fig. 4d and Table 1 clearly suggest the possibility of ET.

However, the IC_50_ values in FRAP assay revealed that AS was higher than RA (Table 1), regardless whether these compounds similarly contain four phenolic –OH groups. This may be because compared with RA, AS has a larger planar conjugation system (i.e. A/C fused rings) to delocalize the positive charges after ET [36]. Our assumption is further supported by the results from the ABTS•^+^ assay, in which AS displayed lower IC_50_ value than RS (Table 1) and a similar trend of dose response curve to that in FRAP assay (Fig. 4e). ABTS•^+^ scavenging, however, was interrupted to comprise a partially reversible ET mechanism [37].

To test the possibility of HAT, we further explored the DPPH•-scavenging capacities of the compounds. As illustrated in Fig. 4f, AS presented a good DPPH•-scavenging activity. The DPPH•-scavenging activity is reported to be affected by various factors, such as pH [38], solvent [39, 40], steric hindering [41], H-bonding [42], and mediated by several types of mechanisms, such as HAT [43], sequential electron proton transfer (SEPT) [43], ET [35], radical adduct formation (RAF) [43], sequential proton loss electron transfer (SPLET) [40], and proton-coupled electron transfer (PCET) [44]). Nevertheless, HAT is regarded as an essential mechanism [43, 45]. Accordingly, DPPH is usually used to evaluate the HAT potential of an antioxidant [46]. The fact that ESG, along with RA and AS, can scavenge DPPH•, suggested that HAT possibly happened to account for the antioxidant activity. However, in this aspect, AS had weaker HAT potential than RA, although both of them contain four phenolic –OH groups (Fig. 2).

**Fig. 5:**
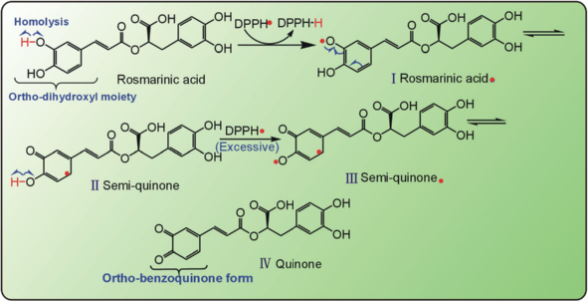
The possible reaction of rosmarinic acid (RA) with DPPH• via hydrogen atom transfer (HAT) pathway

The difference can be attributed to their chemical structures. With the AS molecule, a steric hindrance from the residue of 3-α-L-rhamnose can limit the atom transfer from antioxidant molecule to radical, and H-bonding between 5–OH and 4–C = O may decrease the homolysis of phenolic –OH [41, 42]. However, the above disadvantageous factors for HAT do not occur with the RA molecule. Conversely, the RA molecule bears two moieties with HAT potential. As such, RA can easily undergo HAT pathways to transfer into stable *ortho*-quinone form (Fig. 5).

**Fig. 6:**
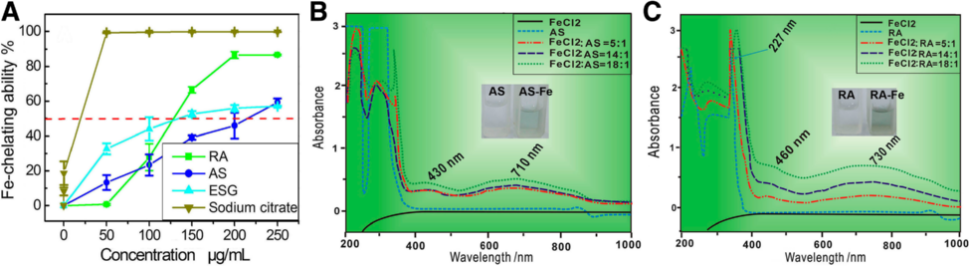
The dose response curve of rosmarinic acid (RA), astilbin (AS) in the Fe^2+^-chelating assay (**a**), the UV spectra of AS-Fe^2+^ complex (**b**), RA-Fe^2+^ complex (**c**). In figure a, each value is expressed as Mean ± SD (*n* = 3). Sodium citrate was used as positive control

As stated in the Methods section, the generation of DPPH• and ABTS•^+^ do not rely on metal catalysis, and therefore, their DPPH• and ABTS•^+^ radical scavenging capacities may be mediated through direct radical-scavenging. On the contrary, the generation of ROS (especially •OH) radical relies on transition metal catalysis (Eq. 1). Hence, Fe^2+^-chelation can decrease the level of •OH radicals and is regarded as indirect •OH radical scavenging [47]. The present study used Ferrozine as the indicator to investigate Fe-chelating abilities. As seen in Fig. 6, ESG, RA, and AS increased Fe^2+^-chelating percentages at 50–250 μg/mL in a concentration-dependent manner. This provides the evidence of Fe^2+^-chelating as an indirect approach for phytophenols to scavenge •OH radicals and then to relieve oxidative stress in MSCs. This is consistent with the result of animal experiment that caffeic acid with catechol moiety could inhibit oxidative stress mediated by iron overload in rats [48]. In fact, some iron chelators (e.g. deferiprone) were reported to be able to completely inhibit the generation of •OH radicals [49].

However, in the case of iron overload, these phenolics could also reduce Fe^3+^ to Fe^2+^, and recycling of Fe^2+^ source that could cause the formation of •OH radicals (Eq. 2). Our results agree with the previous studies that iron reduction potentiates •OH radical formation in flavonols with catechol moiety [50]. The recycling described in Eq. 2 can also explain the metal-dependent pro-oxidant action of gallic acid derivatives or (−)-epigallocatechin-3-gallate (EGCG), which can cause cell apoptosis and formation of 8-hydroxy-2′-deoxyguanosine [51, 52].2


Thereby, under the iron overload and aerobic condition, administration of massive flavonoids may lead to unpredictable consequences [50]. The safest approach may be administration of some iron chelators without reducing power such as deferiprone or deferoxamine [53].

**Fig. 7:**
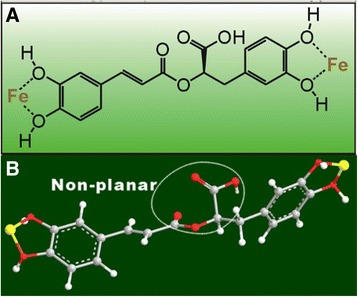
The proposed structure of rosmarinic acid (RA) chelated with Fe^2+^ (**a**), and the ball-stick model of rosmarinic acid (RA, **b**)

In our experiment of Fe^2+^-chelating, we observed a great difference between RA and AS. The colorless RA solution was found to turn green when mixed with FeCl_2_ (Fig. 6b). The RA-Fe^2+^ complex presented an absorption maximum at 730 nm, while RA itself showed an absorption maximum at 227 nm. The great bathochromic shift (λ_max_ 227 → 730 nm) evidently indicates an extension of aromatic conjugation. The metal-chelating has been reported to come from *ortho*- or adjacent –OH and –C = O groups [43]. A possible structure of the RA-Fe^2+^ complex is shown in Fig. 7a. It is noted that, the adjacent –C = O and –COOH groups cannot chelate Fe^2+^, since two groups are non-planar and cannot form a stable ring with Fe^2+^ (Fig. 7b). Compared with RA, AS exhibited lower Fe^2+^-chelating percentages (Fig. 6a). The strength of the absorption maximum of AS-Fe^2+^ complex also became weaker (Fig. 6b and c), while AS-Fe^2+^ complex appeared a bit less green as compared to RA-Fe^2+^ complex (Fig. 6b and c). Quantitative analysis based on IC_50_ values further suggested that the Fe^2+^-chelating level of AS was only 0.72 (355/485) times than that of RA (Table 1).

**Fig. 8:**
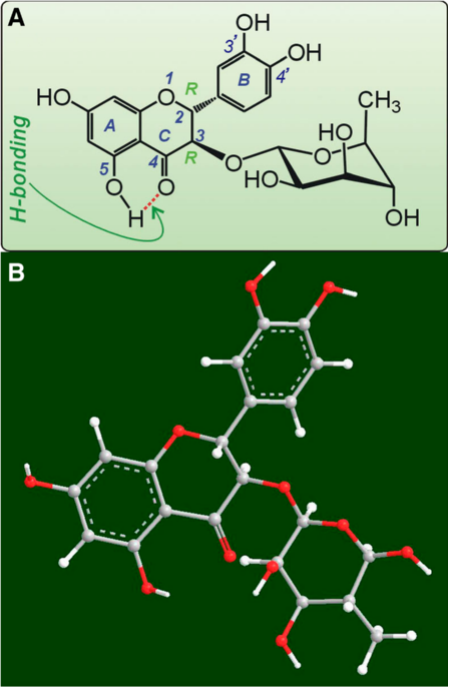
The H-bonding (**a**), and ball-stick model of astilbin (AS) based on preferential conformation (**b**)

Such difference might also be attributed to the chemical structure. Seemingly, in AS molecule, there are two chelating sites, i.e. 3′, 4′-dihydroxy groups (catechol moiety), and between 4–C = O and 5–OH groups (Fig. 8a). However, the H-bonding between 4–C = O and 5–OH groups may hinder the form of Fe^2+^-chelating. Moreover, the steric hindrance from the residue of α-L-rhamnose in 3-position can also reduce the possibility of Fe^2+^-chelating at this site (Fig. 8b).

**Fig. 9:**
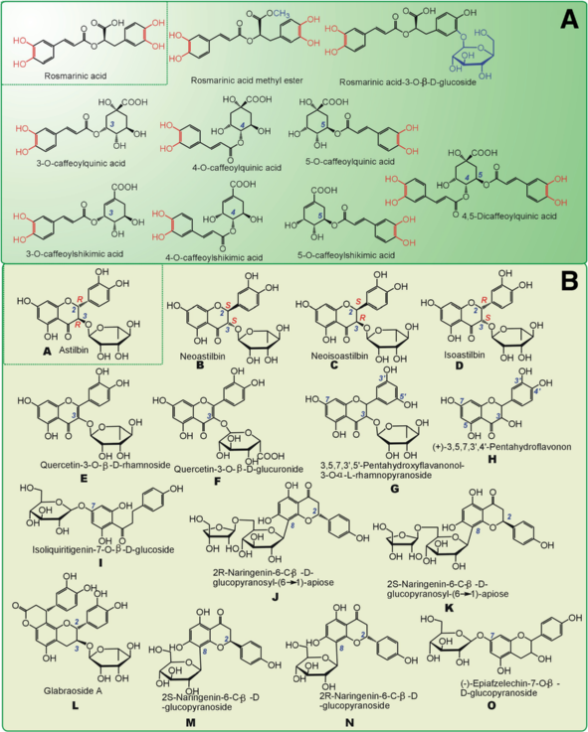
The structures of caffeoyl derivatives (**a**) and flavonoids (**b**) in *S. glabra* [12] (The structure of L glabraoside A is corrected based on [51])

It must be emphasized that, the antioxidant features of RA can be generalized to other caffeoyl derivatives in ESG, because all of these derivatives similarly bear the caffeoyl moieties (Fig. 9a); while those of AS cannot be generalized to other flavonoids because of the difference in chemical structures, such as diflavonone glycosides (A-D, G, J, K, M, N, O), flavonone glycosides (E & F), flavonol (H), chalcone (I), and even flavan lactone (L) (Fig. 9b) [12, 54].

## Conclusion

The traditional Chinese Herb medicine *S. glabra* can protect MSCs from oxidative-stressed apoptosis. Such protective effect can be attributed to its antioxidant ability and to the presence of two kinds of phytophenols, caffeoyl derivatives and flavonoids. As the respective representatives of caffeoyl derivatives and flavonoids, rosmarinic acid and astilbin may exert the antioxidant action via direct ROS-scavenging, and indirect ROS-scavenging (i.e. Fe^2+^-chelating). The direct ROS-scavenging may involve hydrogen atom transfer (HAT) and/or electron transfer (ET) pathways. Astilbin possibly engages the latter pathway due to the larger planar conjugation in A/C fused rings. Rosmarinic acid, on the other hand, presents more HAT and Fe^2+^-chelating ability which can be attributed to rosmarinic acid bearing one more catechol moiety. Astilbin has steric-hindrance from 3-α-L-rhamnose and an H-bonding between 4,5 sites. The antioxidant features of rosmarinic acid can be generalized to other caffeoyl derivatives, while that of astilbin cannot be generalized to other flavonoids because of the difference in chemical structures.

## Additional file

Additional file 1: Experimental protocols for mechanistic chemistry. (DOCX 24 kb)

## Abbreviations

ABTS: [2,2′-azino-bis(3-ethylbenzo-thiazoline-6-sulfonic acid diammonium salt)]
AS: Astilbin
BHA: Butylated hydroxyanisole
DMEM: Dulbecco’s modified Eagle’s medium
DPPH•: (1,1-diphenyl-2-picryl-hydrazl)
ESG: Ethanol extract of *Sarcandra glabra*
ET: Electron transfer
FBS: Fetal bovine serum
HAT: Hydrogen atom transfer
MSCs: Mesenchymal stem cells
MTT: [3-(4,5-dimethylthiazol-2-yl)-2,5-diphenyl]
RA: Rosmarinic acid
ROS: Reactive oxygen species
*S. glabra*: *Sarcandra glabra* (Thunb.) Nakai
SD: Standard deviation
TBA: 2-thiobarbituric acid
TCM: Tradition Chinese Medicine
Tris: Tri-hydroxymethylamino methane
Trolox: [(±)-6-hydroxyl-2,5,7,8-tetramethlychromane-2-carboxylic acid]
