# Supplementary material for: Sarcandra glabra (Caoshanhu) protects mesenchymal stem cells from oxidative stress: a bioevaluation and mechanistic chemistry
Source: BMC Complement Altern Med. 2016 Oct 28;16:423. doi: 10.1186/s12906-016-1383-7 (PMC5084467; doi:10.1186/s12906-016-1383-7)
Supplement: Additional file 1: — Experimental protocols for mechanistic chemistry. (DOCX 24 kb) [file 12906_2016_1383_MOESM1_ESM.docx]

**Addition file 1**

*Sarcandra glabra* (Caoshanhu) protects mesenchymal stem cells from oxidative stress: a bioevaluation and mechanistic chemistry

Jingjing Liu ^a^, Xican Li ^a, *^, Jian Lin ^b^, Yunrong Li^a^, Tingting Wang^a^, Qian Jiang^a^, Dongfeng Chen ^b, *^

^a^ School of Chinese Herbal Medicine, ^b^ School of Basic Medical Science, Guangzhou University of Chinese Medicine, Guangzhou, China, 510006.

^*^Corresponding author. **E-mail:** [lixican@126.com](mailto:lixican@126.com)

**Address:** School of Chinese Herbal Medicine, Guangzhou University of Chinese Medicine, Waihuan East Road No.232, Guangzhou Higher Education Mega Center, 510006, Guangzhou, China.

***Detailed experimental procedure of mechanistic chemistry experiments***

*Scavenging ability of wedelolactone towards •OH radicals (deoxyribose degradation assay)*

The •OH radical-scavenging assay was performed according to the improved deoxyribose degradation method reported in our previous study (Li, 2013). Briefly, each sample was dissolved in ethanol at an appropriate concentration. Aliquots of the sample solutions were collected and placed in mini tubes and evaporated to dryness, before being treated with 10 μL of deoxyribose (50 mM), 20 μL of Na_2_EDTA (1 mM), 10 μL of FeCl_3_ (3.2 mM), 15 μL of H_2_O_2_ (50 mM) and 15 μL of ascorbic acid (1.8 mM). The total volume of each reaction mixture was adjusted to 130 μL with buffer and mixed thoroughly. After incubation at 50 °C for 20 min, each reaction was terminated by the addition of 50 μL of trichloroacetic acid (10%, w/w). The color was then developed by the addition of 30 μL of 2-thiobarbituric acid (5% in a 1.25% NaOH aqueous solution) and heated in an oven at 115 °C for 15 min. The mixture was cooled and its absorbance was measured at 530 nm using a Unico 2100 spectrophotometer (Shanghai, China) against the buffer (as a blank). The •OH radical scavenging activity was expressed as follows:

$Inhibition \% = \frac{A_{0}-A}{A_{0}} \times100\%$.

Where A_0_ and A are the absorbance values of the blank and test samples, respectively.

*Scavenging ability of wedelolactone towards •O_2_^−^ radicals (pyrogallol autoxidation assay)*

The superoxide anion (•O_2_^−^)-scavenging activity was determined using a method previously developed in our laboratory (Li, 2012). Briefly, a 60–300 μL of sample solution (1 mg/mL) was added to Tris-HCl buffer (0.05M, pH 7.4) containing Na_2_EDTA (1 mM) and the total volume was adjusted to 980 μL using buffer. Twenty microliters of pyrogallol solution (60 mM in 1 mM HCl) was added to the sample, and the resulting mixture was vigorously agitated and read at 325 nm every 30 s for 5 min. The •O_2_^−^ radical-scavenging ability was calculated as follows:

.

Where ΔA_325 nm, control_ is the increase in the A_325 nm_ value of the mixture without the sample, ΔA_325 nm, sample_ is the increase in the A_325 nm_ value of the mixture with the sample and T is the determining time (5 min).

[*Colorimetr*](http://www.baidu.com/link?url=S1smmIgdYWub7y5gSOxfvX1NHIO4RxIpWfITc_lkON8jsdq9erww-4c1969MdJAfce-Zll3r5kNE7SsPEk96pxJKGCsOgfgOTqaYatOHfPW)*y determination and ultraviolet (UV) spectra determination of Fe^2+^-chelating*

The Fe^2+^-chelating activity of wedelolactone was estimated using a [colorimetr](http://www.baidu.com/link?url=S1smmIgdYWub7y5gSOxfvX1NHIO4RxIpWfITc_lkON8jsdq9erww-4c1969MdJAfce-Zll3r5kNE7SsPEk96pxJKGCsOgfgOTqaYatOHfPW)ic method (Gülçn, 2012). Briefly, a 0–100-μL aliquot of sample solution (1 mg/mL in methanol) was added to 100 μL of an aqueous FeCl_2_•4H_2_O solution (250 μM), and the resulting mixture was treated with 150 μL of aqueous ferrozine (1 mM) to initiate the reaction. The total volume of the system was then adjusted to 1000 μL with methanol, and the resulting mixture was vigorously shaken for 5 min before being allowed to stand at room temperature for 10 min. The absorbance of the solution was then recorded at 562 nm (Jinhua 754 PC, Shanghai, China). The percentage chelation activity was calculated using the formula:

$$Inhibition \% = \frac{A_{0}-A}{A_{0}} \times100\%$$

The wedelolactone-Fe^2+^ complex was evaluated by UV spectroscopy. For these experiments, 200 μL of a methanolic solution of wedelolactone (1 mg/mL) was added to 300 μL of an aqueous solution of FeCl_2_•4H_2_O (5 mg/mL). The resulting mixture was subjected to ultrasonic radiation before being centrifuged at 6500 *×g* for 10 min. The supernatant was collected and analyzed on a UV/Vis spectrophotometer (Jinhua 754 PC, Shanghai, China).

*ABTS•^+^ radical scavenging assay*

The ABTS•^+^ scavenging activity was evaluated using a modified version of a previously reported method (Herraiz et al., 2004). ABTS•^+^ was produced by mixing 200 μL of ABTS diammonium salt (7.4 mM) with 200 μL of K_2_S_2_O_8_ (2.6 mM). After being incubated in the dark for 12 h, the mixture was diluted with methanol (about 1:50, v/v) until its absorbance at 734 nm was 0.70±0.02. The diluted ABTS^+^• solution (800 μL) was then treated with 200 μL of ethanol containing different concentrations of wedelolactone and thoroughly mixed. The reaction mixture was then left to stand for 6 min and its absorbance was read at 734 nm on a spectrophotometer. The percentage inhibition was calculated using the formula:

$$Inhibition \% = \frac{A_{0}-A}{A_{0}} \times100\%$$

*Cupric ions (Cu^2+^) reducing power assay*

A Cu^2+^-reducing power assay was carried out according to a previously reported method (Li et al., 2012a). Briefly, 125 μL of aqueous CuSO_4_ (10 mM) was mixed with 125 μL of neocuproine solution (7.5 mM in CH_3_OH), followed by solutions containing different concentrations of wedelolactone. The total volume of each mixture was then adjusted to 1000 μL with CH_3_COONH_4_ buffer and mixed vigorously. The absorbance of each solution was then measured against a buffer blank at 450 nm after 30 min. The relative Cu^2+^-reducing power was calculated using the following formula:

$Relative reducing power \% = \frac{A-A_{min}}{A_{max}-A_{min}} \times100\%$.

Where A_max_ and A_min_ are respectively the maximum and minimum absorbance values in each test and A is the absorbance of the test sample.

*DPPH*• *radical scavenging assay*

The DPPH• radical-scavenging ability was assessed according to the method reported by Li (Li et al., 2012b). Briefly, 500 μL of a methanolic solution (at least five different concentrations were prepared) was mixed with 1000 μL of DPPH• solution (100 μM in methanol, prepared daily). The mixture was shaken vigorously and left to stand for 30 min in the dark. The absorbance of the mixture was then measured at 519 nm against a blank. The DPPH• radical-scavenging activity of each solution was calculated as a percentage inhibition, according to the equation:

$$Inhibition \% = \frac{A_{0}-A}{A_{0}} \times100\%$$
